# Subspace Compressive GLRT Detector for MIMO Radar in the Presence of Clutter

**DOI:** 10.1155/2015/341619

**Published:** 2015-10-01

**Authors:** Siva Karteek Bolisetti, Mohammad Patwary, Khawza Ahmed, Abdel-Hamid Soliman, Mohamed Abdel-Maguid

**Affiliations:** ^1^Sensing, Processing & Communications Laboratory, Faculty of Computing, Engineering & Sciences, Staffordshire University, Stoke-on-Trent ST4 2DE, UK; ^2^School of Science and Engineering, United International University, Dhanmondi, Dhaka 1209, Bangladesh; ^3^School of Science, Technology and Health, University Campus Suffolk, Ipswich IP4 1QJ, UK

## Abstract

The problem of optimising the target detection performance of MIMO radar in the presence of clutter is considered. The increased false alarm rate which is a consequence of the presence of clutter returns is known to seriously degrade the target detection performance of the radar target detector, especially under low SNR conditions. In this paper, a mathematical model is proposed to optimise the target detection performance of a MIMO radar detector in the presence of clutter. The number of samples that are required to be processed by a radar target detector regulates the amount of processing burden while achieving a given detection reliability. While Subspace Compressive GLRT (SSC-GLRT) detector is known to give optimised radar target detection performance with reduced computational complexity, it however suffers a significant deterioration in target detection performance in the presence of clutter. In this paper we provide evidence that the proposed mathematical model for SSC-GLRT detector outperforms the existing detectors in the presence of clutter. The performance analysis of the existing detectors and the proposed SSC-GLRT detector for MIMO radar in the presence of clutter are provided in this paper.

## 1. Introduction

A radar system is expected to search for designated targets within a given region by detecting the existence of the reflected components of that transmitted signal from the target. For any radar system, signal detection is the primary and the most important process. In the existing literature, different signal detection models such as Generalised Likelihood Ratio Test (GLRT) detector [1, 2] and Rao test detector [3, 4] have been widely considered for their robustness. A GLRT detector has attracted the interest of the researchers due to its robustness, simplicity, and ability to display constant false alarm rate (CFAR). Target detection performance of a radar system can be significantly affected by variations in target Radar Cross-Section (RCS). With Multiple Input and Multiple Output (MIMO) antenna connections at the radar system, it is possible to assure the existence of spatiotemporal nature within the received signal. Subsequently, MIMO radars obtained significant research attention within the relevant research community [5–9]. In MIMO radars, the received signal components from different transmitter-receiver pairs are statistically uncorrelated. By exploiting the uncorrelated nature of spatiotemporal received signals, the effect of variation of target RCS can be optimised. In the existing literature, authors have addressed different target detection problems related to MIMO radars and proposed solutions to enhance the target detection performance [10, 11]. The multiple transmitters and receivers of MIMO radars can either be collocated or widely spaced. MIMO radars with widely spaced antennas are capable of achieving improved spatial diversity with respect to the target radar cross-section. On the other hand MIMO radars with collocated antennas are capable of providing improved waveform diversity and increased accuracy in signal parameter estimation. However, diversity of the signal statistics at the multiple receivers of a MIMO radar is achieved at the cost of increased processing burden on the radar system. For a radar system, the time taken by its detector to make a decision is relative to the number of received signal statistics which are required to be processed. The large number of signal statistics collected by the multiple receiver antennas of MIMO radars imposes huge processing burden and computational complexity on the system. Compressive sampling has been addressed in [8, 12–19] where the detection process is performed on compressed received signal samples. The authors in [15] have proposed compressive detection for MIMO radars to reduce the processing complexity which is achieved as a trade-off with the target detection performance. By exploiting the target sparsity in the Doppler range, better target detection performance can be achieved with a fewer number of received signal samples. A relatively new compressive signal realisation technique called subspace compression has been proposed in [20]. The authors in [8, 14] addressed a GLRT detector known as Compressive GLRT (C-GLRT) detector, which performs radar target detection over compressed received signal samples. By using compressed received signal samples, a C-GLRT detector has the ability to be faster and operate at reduced computational complexity. However, these traits are achieved as a trade-off between the radar target detection performance and signal compressibility. For an intended probability of detection, the degree of compressibility gets poorer due to the time varying nature of target detection environment. A time adaptive compressive measurement scheme has been presented within the subspace of a Gaussian measurement matrix, named SSC scheme in [14, 20]. As subspace compressive measurement scheme takes control over this measurement matrix which is adaptive to the signal subspace characteristics. A Subspace Compressive GLRT (SSC-GLRT) detector is expected to give better target detection performance compared to a C-GLRT detector.

Clutter is comprised of all the reflected return signals from the extraneous background environment that arrive at the radar detector. Clutter returns appear on the same domain as the target returns. The presence of clutter is known to cause increased false alarms and hence compromise the target detection performance of the radar detector at a constant false alarm rate [21]. The deterioration in target detection performance is significantly increased when compressive sampling is used. In the existing literature, authors proposed using Doppler shift caused due to moving targets to negotiate clutter [22, 23]. While this approach yields performance gains in the case of fast moving targets and airborne radars, alternative approaches need to be investigated for ground based radars with slow moving targets.

In most of the existing research, the presence of clutter has not been addressed by the authors in the context of Compressive GLRT techniques. The main contribution of this paper is providing a detailed mathematical model to optimise the target detection performance of C-GLRT and SSC-GLRT detectors for a MIMO radar detector in the presence of clutter by exploiting the known knowledge of the clutter subspace. The proposed mathematical model is applied to Compressive and Subspace Compressive GLRT detection schemes and the corresponding target detection performance gains are measured. The target detection probabilities of the proposed and conventional GLRT detectors in the presence of clutter are plotted to demonstrate the superiority of the proposed model. The proposed compressing sensing techniques find their applications in resource constrained security applications with limited processing capabilities. The rest of the paper is organised as follows. In Section 2, the system model and the signal models for binary hypothesis testing are introduced. In Section 3, the test statistic for a GLRT detector in the presence of clutter is derived. In Section 4, the proposed mathematical model for SSC-GLRT detector in the presence of clutter is derived. The performance evaluation and simulation results are given in Section 5. Conclusions and future work are summarised in Section 6.

## 2. Signal Model and Hypothesis Testing

### 2.1. Signal Model

**Figure 1:**
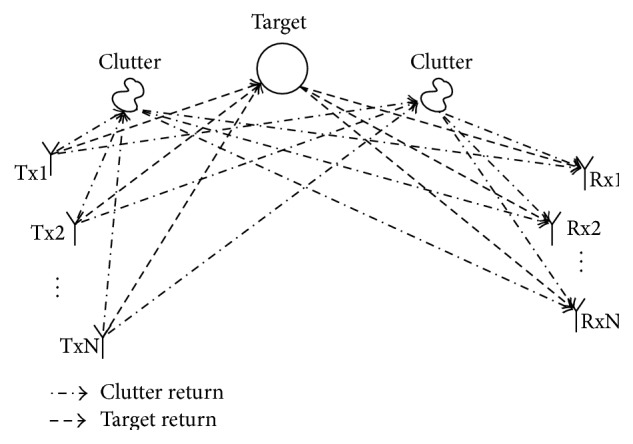
System model for a bistatic MIMO radar.

As aforementioned, the problem of interest which is considered in this paper is detecting the presence of a target using ground based bistatic MIMO radar. The MIMO radar is assumed to have *N*_*t*_ transmitting antennas and *N*_*r*_ receiving antennas. Each receiving antenna is assumed to have *N*_*a*_ array elements. It is assumed that each transmitting antenna transmits *N*_*p*_ coherent pulses per transmitting cycle (Figure 1).

In the presence of a target within a cluttered background, the received signal at each receiver element can be expressed as a combination of target return, clutter return, and noise. Hence the received signal at each MIMO receiver can be mathematically modelled as

(1) yi=Sai+Hbi+wi,ai=a1,a2,…,aNtT,i=1,2,…,Nr,bi=b1,b2,…,bNtT,i=1,2,…,Nr,

where **y**_*i*_ is the received signal at the *i*th receiver antenna and it is of dimensions (*N*_*p*_ *N*_*a*_ × 1), **S** is the steering vector of dimensions (*N*_*p*_ *N*_*a*_ × *N*_*t*_), **H** represents the clutter subspace and is of dimensions (*N*_*p*_ *N*_*a*_ × *N*_*t*_), **a**_*i*_ is the unknown complex value accounting for target backscattering power and channel propagation between transmitter, target, and the receiver and it is of dimension (*N*_*t*_ *N*_*t*_ × 1), and **b**_*i*_ is the unknown complex amplitude of the clutter return which is of dimensions (*N*_*t*_ × 1). Finally, **w**_*i*_ denotes the noise component which is of dimensions (*N*_*p*_ *N*_*a*_ × 1).

In (1), the clutter subspace matrix **H** is a priori unknown. Detection algorithms suffer deterioration in the detection performance in the presence of unknown clutter. The knowledge of clutter is necessary to achieve reliable detection rates and hence clutter estimation is necessary prior to target detection. To estimate clutter, the knowledge of a set of *K* secondary data which are free of target returns is necessary:

(2) $$\begin{array}{c} y_{i,k}=Hb_{i}+w_{i,k},\;k=1,2,\ldots ,K. \end{array}$$

In the existing literature, the authors have addressed the problem of clutter estimation from the available secondary data [24–27]. For the rest of this paper, it is assumed that a reliable clutter estimate is available to the target detector with clutter being relatively time invariant.

### 2.2. Hypothesis Testing

The performance measure of a radar receiver, while being dedicated to detect the existence or nonexistence of targets within a region of interest, is the degree of reliability on such decision making. The two possible outcomes of this decision making process are occurrence or nonoccurrence of a phenomenon representing existence and nonexistence of the target, respectively, which is modelled as a binary hypothesis testing problem. The two possible hypotheses are *H*_0_ and *H*_1_, where *H*_0_ represents the absence of the target and *H*_1_ represents the presence of the target. The corresponding signal models of these hypotheses are [14, 21]

(3) H0:y=Hb+w,H1:y=Sa+Hb+w.

The amplitude vector **a** and the noise variance are assumed to be unknown to the radar receiver, while noise is assumed to be AWGN. The test statistic for the GLRT detector is generated from the log-likelihood ratio function within which the unknown parameters are estimated using Maximum Likelihood (ML) estimator. For a desired false alarm rate (*P*_fa_), a threshold *γ* is generated which is compared with the likelihood ratio function such that a decision regarding the presence or absence of the target can be made.

## 3. GLRT Detector in the Presence of Clutter

Clutter signal returns are spread across frequency spectrum and away from zero frequency. Clutter returns are often known to lead to increased false alarm rates. With relatively small target Radar Cross-Sections (RCS), it is often the case where the signal strengths from target returns are weaker than the clutter returns and hence makes target detection process more difficult at a constant false alarm rate (CFAR). Hence careful considerations of the effect of clutter returns are to be included in target detection design process to maintain the required CFAR. For the received signal models described in (3), the joint probability density functions for the unknown parameters under hypotheses *H*_0_ and *H*_1_ are defined as

(4) fy ∣ b,σ2,H0=1πσ2N·exp⁡−1σ2y−HbHy−Hb+∑k=1KykHyk,fy ∣ a,b,σ2,H1=1πσ2N·exp⁡−1σ2y−Sa−HbHy−Sa−Hb+∑k=1KykHyk.

It is assumed that the radar target detector does not have the knowledge of the noise variance, represented by **σ** ^2^, and the complex amplitudes of clutter and target returns which are represented by **b** and **a**, respectively. To formulate the test statistic, the unknown parameters are estimated by maximising the unknown parameter values for a given set of received signal samples. The Maximum Likelihood (ML) estimator estimates these unknown parameters from the log-likelihood function which is denoted by Γ. The log-likelihood functions under hypotheses *H*_0_ and *H*_1_ are summarised as

(5) Γy ∣ b,σ2,H0=−Nlog⁡πσ2−1σ2y−HbHy−Hb+∑k=1KykHyk,

(6) Γy ∣ a,b,σ2,H1=−Nlog⁡πσ2−1σ2y−Sa−HbHy−Sa−Hb+∑k=1KykHyk.

### 3.1. ML Estimate of Noise Variance

Let the ML estimates of the noise variance, **σ** ^2^, under hypotheses *H*_0_ and *H*_1_ be denoted by ${\hat{\sigma }}_{0}^{2}$ and ${\hat{\sigma }}_{1}^{2}$, respectively. The corresponding ML estimates can be obtained from the partial derivatives of (5) and (6) with respect to **σ** ^2^:

(7) ∂∂σ2Γy:b,σ2,H0=0,

(8) ∂∂σ2Γy:a,b,σ2,H1=0,

and thus, by solving (7) and (8), ML estimates of **σ** ^2^ under hypotheses *H*_0_ and *H*_1_ can be summarised as

(9) σ^02=1Ny−HbHy−Hb+∑k=1KykHyk,

(10) σ^12=1Ny−Sa−HbHy−Sa−Hb+∑k=1KykHyk.

### 3.2. ML Estimate of Clutter Return

Let the ML estimates of the unknown complex amplitude of the clutter signal return under hypotheses *H*_0_ and *H*_1_ be denoted by ${\hat{b}}_{0}$ and ${\hat{b}}_{1}$, respectively. As aforementioned, the corresponding ML estimates can be obtained from the partial derivatives of (5) and (6) with respect to **b**:

(11) ∂∂bΓy:b,σ2,H0=0,

(12) ∂∂bΓy:a,b,σ2,H1=0.

Solving (11) and (12), it can be observed that ML estimate of the complex amplitude of the clutter signal return is independent of **σ** ^2^. ML estimates of **b** under hypotheses *H*_0_ and *H*_1_ can be summarised as

(13) b^0=HHH−1HHy,

(14) b^1=HHH−1HHy−Sa.

### 3.3. ML Estimate of Target Return

The complex amplitude of the radar signal which is backscattered from the target is unknown to the radar detector. Let the ML estimate of the target return under hypotheses *H*_0_ and *H*_1_ be denoted by ${\hat{a}}_{0}$ and ${\hat{a}}_{1}$, respectively. From (5) and (6),

(15) ∂∂aΓy:b,σ2,H0=0,

(16) ∂∂aΓy:a,b,σ2,H1=0.

Hypothesis *H*_0_ is based on the assumption that there is no target return. From (16) it can be observed that the ML estimate of the target return ${\hat{a}}_{0}$ under hypothesis *H*_0_ is

(17) $$\begin{array}{c} {\hat{a}}_{0}=0. \end{array}$$

ML estimate of ${\hat{a}}_{1}$ can be obtained from (16) and (14) as

(18) ∂∂ay−Sa−HHHH−1HHy−SaH·y−Sa−HHHH−1HHy−Sa=0.

Therefore the ML estimate of complex amplitude of the target return can be summarised by solving (18) as

(19) $$\begin{array}{c} {\hat{a}}_{1}={\left(\;S^{H}\left(\;P^{H}P\;\right)S\;\right)}^{-1}\left(\;S^{H}\left(\;P^{H}P\;\right)y\;\right), \end{array}$$

where **P** = **I** − **H**(**H** ^*H*^ **H**)^−1^ **H** ^*H*^.

By using the ML estimates of the unknown parameters, the test statistic can be obtained as

(20) $$\begin{array}{c} \zeta =\frac{{\left(y-H{\hat{b}}_{0}\right)}^{H}\left(y-H{\hat{b}}_{0}\right)+\sum y_{k}^{H}y_{k}}{{\left(y-S\hat{a}-H{\hat{b}}_{1}\right)}^{H}\left(y-S\hat{a}-H{\hat{b}}_{1}\right)+\sum y_{k}^{H}y_{k}}. \end{array}$$

## 4. Proposed Subspace Compressive GLRT Detector in the Presence of Clutter

In Section 3, we derived the test statistic for a GLRT detector for MIMO radar to detect the presence of a target using a given set of received signal samples in the presence of clutter. The processing requirement within a radar receiver is a function of the number of targets that are required to be dissociated from the given set of received signal samples. In other words, to provide a preset level of target detection reliability, the required number of received signal samples varies nonlinearly with the number of targets that are required to be dissociated. With limited computational capacity within a radar receiver, such increase in processing complexity may lead towards resource saturation, hence leading towards a trade-off with the target detection reliability. To reduce the processing burden, C-GLRT has been proposed by authors in the existing literature. In C-GLRT, the received signal samples are compressed by projecting them onto a projection matrix Φ. While C-GLRT has the ability to make a decision over existence or nonexistence of the target based on compressed received signal samples and hence reducing the computational complexity, it however suffers a significant deterioration in the target detection performance. Moreover, the target detection performance is further deteriorated in the presence of clutter. Subspace compression techniques are known to give better trade-off between the performance and compressibility when compared to conventional compression techniques. Hence a SSC-GLRT is expected to give a better target detection performance than a C-GLRT. The signal subspace for the radar target returns is expected to be sparse in nature. The projection matrix for SSC-GLRT is modelled to exploit this sparse nature of the received signal samples. In this section, we derive a new mathematical framework to obtain the test statistic for SSC-GLRT detector which is expected to improve the target detection performance in the presence of clutter.

### 4.1. Signal Model

Unlike a C-GLRT which uses a random projection matrix to compress the received signal samples, for SSC-GLRT, we derive the projection matrix based on the knowledge of the signal subspace. The projection matrix Φ for SSC-GLRT can be derived as

(21) $$\begin{array}{c} \Phi =G{\left(\;S^{T}S\;\right)}^{-1}S^{T}, \end{array}$$

where **G** is the random measurement matrix.

For SSC-GLRT, the compressed received signal model under hypotheses *H*_0_ and *H*_1_ can be obtained from (3) and (21) as

(22) H0:y¯=ΦHb+Φw,H1:y¯=ΦSa+ΦHb+Φw.

SSC-GLRT detector uses the received signal models as described in (22) to make a decision regarding the existence or nonexistence of a target. As mentioned in Section 3, the unknown parameters are statistically estimated using ML estimator. The joint probability density functions for the unknown parameters for SSC-GLRT under hypotheses *H*_0_ and *H*_1_ are defined as

(23) fy¯:b,σ2,H0=1πσ2Nexp⁡−1σ2y¯−ΦHbH·ΦΦH−1y¯−ΦHb+∑y¯kHΦΦH−1y¯k,fy¯:a,b,σ2,H1=1πσ2N·exp⁡−1σ2y¯−ΦSa−ΦHbHΦΦH−1·y¯−ΦSa−ΦHb+∑y¯kHΦΦH−1y¯k.

While the measurement matrix Φ is known to the radar detector, the noise variance and the complex amplitudes of the clutter and target returns are the unknown parameters. For the probability density functions as defined in (23), the log-likelihood functions for SSC-GLRT under hypotheses *H*_0_ and *H*_1_ are expressed as

(24) Γy¯:a,b,σ2,H0=−Nlog⁡πσ2−1σ2Φ−1y¯−ΦHbHΦ−1y¯−ΦHb+∑y¯kHΦΦH−1y¯k,

(25) Γy¯:a,b,σ2,H1=−Nlog⁡πσ2−1σ2Φ−1y¯−ΦSa−ΦHbH·Φ−1y¯−ΦSa−ΦHb+∑y¯kHΦΦH−1y¯k.

### 4.2. ML Estimate of Noise Variance

Let the ML estimates of the noise variance **σ** ^2^ under hypotheses *H*_0_ and *H*_1_ be denoted by ${\hat{\sigma }}_{0}^{2}$ and ${\hat{\sigma }}_{1}^{2}$, respectively. The corresponding ML estimates can be obtained from the partial derivatives of (24) and (25) with respect to **σ** ^2^:

(26) ∂∂σ2Γy¯:b,σ2,H0=0,

(27) ∂∂σ2Γy¯:a,b,σ2,H1=0.

Solving (24), (25), (26), and (27), ML estimates for **σ** ^2^ under hypotheses *H*_0_ and *H*_1_ can be summarised as

(28) σ^02=1NΦ−1y¯−ΦHbHΦ−1y¯−ΦHb+∑y¯kHΦΦH−1y¯k,

(29) σ^12=1NΦ−1y¯−ΦSa−ΦHbH·Φ−1y¯−ΦSa−ΦHb+∑y¯kHΦΦH−1y¯k.

### 4.3. ML Estimate of Clutter Return

The ML estimates of the complex amplitude of the clutter signal returns under hypotheses *H*_0_ and *H*_1_ which are denoted by ${\hat{b}}_{0}$ and ${\hat{b}}_{1}$ can be obtained from the partial derivatives of (24) and (25) with respect to **b**:

(30) ∂∂bΓy¯:b,σ2,H0=0,

(31) ∂∂bΓy¯:a,b,σ2,H1=0.

Solving (30) and (31) and rearranging terms, we can obtain the ML estimates ${\hat{b}}_{0}$ and ${\hat{b}}_{1}$ as

(32) b^0=Vy¯,

(33) b^1=Vy¯−ΦSa,

where **V** = ((Φ**H**)^*H*^(ΦΦ^*H*^)^−1^(Φ**H**))^−1^(Φ**H**)^*H*^(ΦΦ^*H*^)^−1^.

### 4.4. ML Estimate of Target Return

Target return is the energy gathered by the radar receiver which is backscattered from a target. Hypothesis *H*_0_ is based on the assumption that the target is absent. Hence, under *H*_0_, the complex amplitude of the target return has zero magnitude. However, under hypothesis *H*_1_, the ML estimate of the target return which is denoted by ${\hat{a}}_{1}$ can be obtained as

(34) $$\begin{array}{c} \frac{\partial }{\partial a}\left(\;\Gamma \left(\;\bar{y}:a,b,\sigma^{2},H_{1}\;\right)\;\right)=0. \end{array}$$

Solving (34) as aforementioned, the ML estimate of the target return ${\hat{a}}_{1}$ for SSC-GLRT detector in the presence of clutter can be obtained as

(35) $$\begin{array}{c} {\hat{a}}_{1}={\left(\;{\left(\;\Phi S\;\right)}^{H}P\left(\;\Phi S\;\right)\;\right)}^{-1}{\left(\;\Phi S\;\right)}^{H}P\bar{y}, \end{array}$$

where **P** = ((**I** − Φ**H** **V**)^*H*^(ΦΦ^*H*^)^−1^(**I** − Φ**H** **V**)).

By using the ML estimates of the unknown parameters, the test statistic can be obtained from (28) and (29) as

(36) $$\begin{array}{c} \zeta =\frac{{\left(\bar{y}-\Phi H{\hat{b}}_{0}\right)}^{H}{\left(\Phi \Phi^{H}\right)}^{-1}\left(\bar{y}-\Phi H{\hat{b}}_{0}\right)+\sum {\bar{y}}_{k}^{H}{\left(\Phi \Phi^{H}\right)}^{-1}{\bar{y}}_{k}}{{\left(\bar{y}-\Phi S\hat{a}-\Phi H{\hat{b}}_{1}\right)}^{H}{\left(\Phi \Phi^{H}\right)}^{-1}\left(\bar{y}-\Phi S\hat{a}-\Phi H{\hat{b}}_{1}\right)+\sum {\bar{y}}_{k}^{H}{\left(\Phi \Phi^{H}\right)}^{-1}{\bar{y}}_{k}}. \end{array}$$

## 5. Simulations

**Figure 2:**
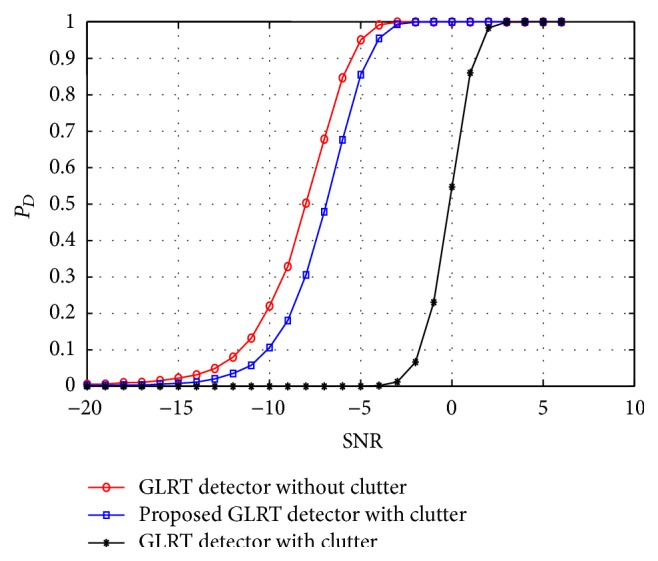
*P*_*D*_ comparison of a conventional GLRT detector and the proposed GLRT detector in the presence of clutter.

**Figure 3:**
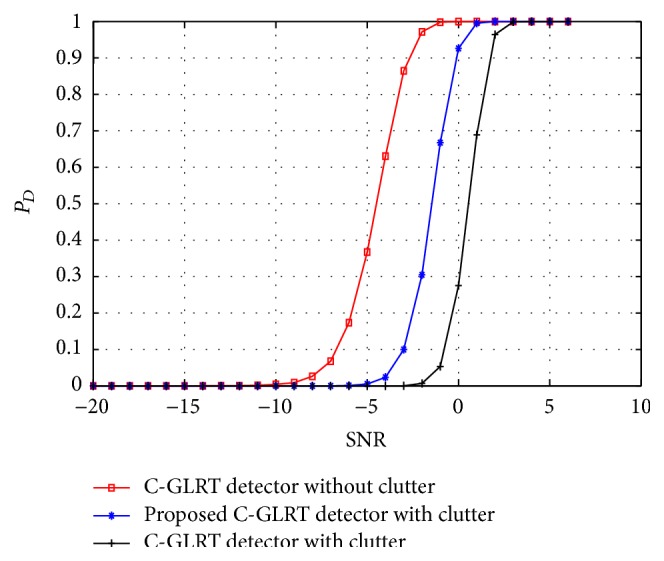
*P*_*D*_ comparison of a conventional C-GLRT detector and the proposed C-GLRT detector in the presence of clutter at 50% compression.

**Figure 4:**
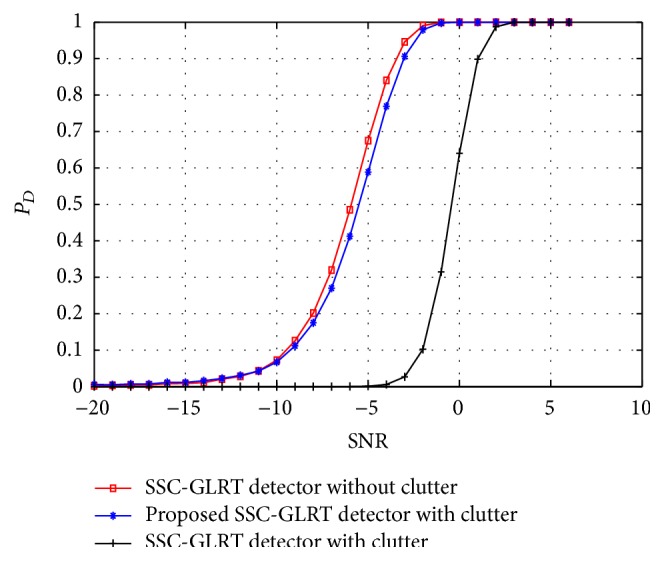
*P*_*D*_ comparison of a conventional SSC-GLRT detector and the proposed SSC-GLRT detector in the presence of clutter at 80% compression.

**Figure 5:**
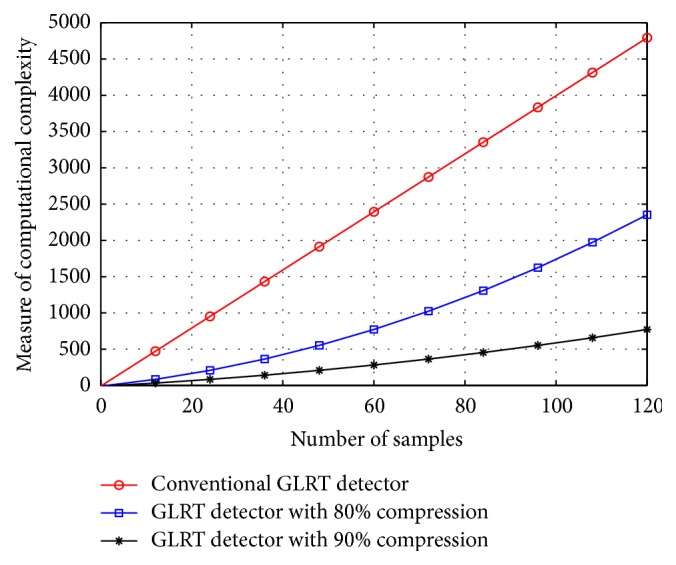
Comparison of computational complexities at different compression ratios.

In this section, we demonstrate the performance of the proposed mathematical model for a MIMO radar target detector in the presence of clutter. As a measure of radar target detection performance we denote the terms probability of detection (*P*_*D*_) which is defined as the percentage of cases in which the true presence of targets is detected and Probability of False Alarm (*P*_fa_) which is defined as the percentage of cases in which the presence of targets is falsely assumed. The experiments are conducted based on Monte Carlo simulations averaged over 10000 samples. A ground based bistatic MIMO radar is considered with *N*_*t*_ = 1 transmitting antenna and *N*_*r*_ = 3 receiving antennas. It is assumed that each receiving antenna has *N*_*a*_ = 4 array elements and the transmitting antennas transmit *N*_*p*_ = 5 coherent pulses per transmitting cycle. The received signal samples are considered to be corrupted by clutter and noise. While noise is assumed to be zero-mean Gaussian, clutter is assumed to follow Rayleigh distribution. Simulations are conducted under CFAR with *P*_fa_ maintained at 10^−4^. In Figure 2, the target detection performance of the conventional GLRT and the proposed GLRT detectors in the presence of clutter is plotted. The performance of the GLRT detector in the absence of clutter is also plotted for comparative reasons. A clear loss of target detection performance for a conventional GLRT detector in the presence of clutter can be observed from the figure while the proposed GLRT detector demonstrated a significant improvement in the target detection performance. Similarly in Figures 3 and 4 the target detection performance of the proposed C-GLRT and SSC-GLRT detectors is plotted. While conventional SSC-GLRT detectors are known to reduce the computational complexity of the radar detector while providing target detection performances which are comparable to conventional GLRT detectors, however, when tested in the presence of clutter, a severe loss of target detection performance has been observed. From Figures 3 and 4 it can be clearly observed that our proposed C-GLRT and SSC-GLRT detectors achieve significantly higher target detection rates with reduced computational complexities. In Figure 5 the computational complexities of a conventional GLRT detector and the proposed SSC-GLRT detector are compared. The computational complexities are measured as a function of the number of arthematic operations involved for a given set of received signal samples during the target detection process.

## 6. Conclusion

In this paper, we have proposed a novel mathematical model to optimise the target detection performance of a MIMO radar in the presence of clutter. A GLRT detector is known to provide robust performance. The proposed mathematical model is tested on the conventional GLRT detector in the presence of clutter and a significant improvement in the target detection performance has been observed. A GLRT detector however requires a large number of received signal samples to provide optimal detection performance at CFAR. Compressive sensing for GLRT detector has been investigated to reduce the computational complexity of the target detector. From the simulation results, it can be clearly observed that a C-GLRT detector, while reducing the computational complexity, also suffers a significant loss of target detection performance. C-GLRT detector is tested in the presence of clutter and a further deterioration in the target detection performance has been observed. Our proposed mathematical model, when tested on C-GLRT detector, produced a significant improvement in the target detection performance. However, a SSC-GLRT detector in known to provide superior target detection performance when compared to C-GLRT detector. Hence, a SSC-GLRT detector has been tested in the presence of clutter and our proposed mathematical model has been applied to produce a clear improvement in target detection performance. Results are plotted for each of the three aforementioned detectors where the ideal performance, performance in the presence of clutter, and performance of the proposed model in the presence of clutter can be compared. It can be clearly observed that our proposed model provides significant performance gains in each of the three cases. Dynamic clutter suppression for SSC-GLRT detector is believed to provide better performance if added as signal preprocessing which we intend to investigate in our future work.
